# Syphilitic Coronary Artery Ostial Stenosis Resulting in Acute Myocardial Infarction Treated by Percutaneous Coronary Intervention

**DOI:** 10.1155/2010/830583

**Published:** 2010-11-01

**Authors:** Marcelo A. Nakazone, Maurício N. Machado, Raphael B. Barbosa, Márcio A. Santos, Lilia N. Maia

**Affiliations:** ^1^Department of Cardiology and Cardiovascular Surgery, São José do Rio Preto Medical School, 15090-000 São José do Rio Preto, SP, Brazil; ^2^Department of Molecular Biology, São José do Rio Preto Medical School, Avenue Brigadeiro Faria Lima 5416, 15090-000 São José do Rio Preto, SP, Brazil

## Abstract

Cardiovascular abnormalities are well-known manifestations of tertiary syphilis infections which although not frequent, are still causes of morbidity and mortality. A less common manifestation of syphilitic aortitis is coronary artery ostial narrowing related to aortic wall thickening. We report a case of a 46-year-old male admitted due to acute anterior ST elevation myocardial infarction submitted to primary percutaneous coronary intervention successfully. Coronary angiography showed a suboccluded ostial lesion of left main coronary artery. VDRL was titrated to 1/512. The patient was discharged with treatment including benzathine penicillin. Previous case reports of acute myocardial infarction in association with syphilitic coronary artery ostial stenosis have been reported, but the fact that the patient was treated by percutaneous coronary intervention is unique in this case.

## 1. Introduction

Syphilitic obliteration of the coronary ostia is an uncommon manifestation of tertiary syphilis infection [1]. Cardiovascular syphilis should be considered in cases of coronary artery ostial lesion with a normal distal bed [2]. This paper describes a case of a 46-year-old male without risk factors for atherosclerosis admitted with acute anterior ST elevation myocardial infarction treated by percutaneous coronary intervention in a patient with syphilitic coronary artery ostial stenosis.

## 2. Case Report

 In June 2010, a 46-year-old male presented to the emergency department with sudden onset of severe chest pain, associated to progressive dyspnea and diaphoresis. He was a smoker but had no other risk factors for coronary artery disease. He had never received radiotherapy or chemotherapy. The patient was tachypneic at 26 breaths/min at admission, with regular rhythm at 160 beats/min. His blood pressure was 130/80 mmHg, and oxygen saturation measured through pulse oximetry was 83%. Cardiac auscultation was normal, and crackles were heard over lungs. The patient was submitted to orotracheal intubation and invasive mechanical ventilation considering acute pulmonary edema. 

**Figure 1:**
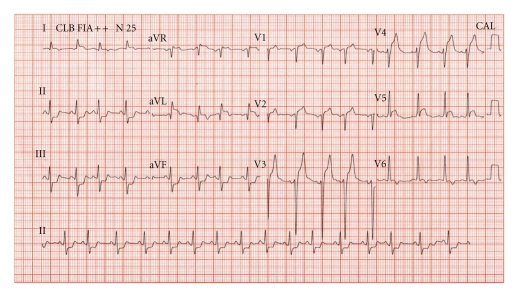
Electrocardiography showing sinus rhythm with ST elevation in leads I, aVL, aVR, V1 to V5 and ST depression in leads DII, DIII, and aVF, compatible with anterolateral wall acute myocardial infarction.

**Figure 2:**
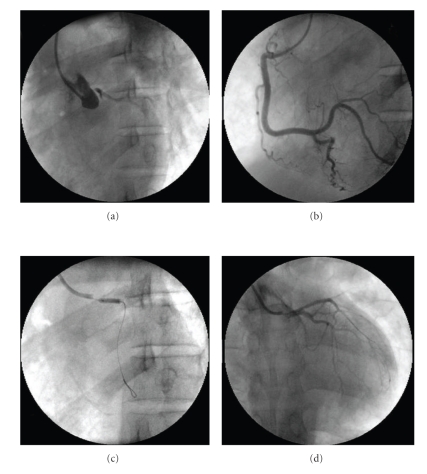
Coronary angiogram showing: (a) a suboccluded ostial lesion of left main coronary artery; (b) a normal right coronary artery; (c) balloon inflation and stent release during the percutaneous coronary intervention; (d) final result with angiographic success.

Electrocardiography showed sinus rhythm with ST elevation in leads I, aVL, aVR, V1 to V5 and ST depression in leads DII, DIII, and aVF, compatible with anterolateral wall acute myocardial infarction (Figure 1). Acetylsalicylic acid and clopidogrel were administered, and the patient was routed to the catheterization laboratory. Coronary angiography showed a suboccluded ostial lesion of left main coronary artery (Figure 2) with a normal distal bed and subtle aortic regurgitation. The percutaneous coronary intervention with stent was promptly and successfully attempted. During this procedure, the patient presented signals compatible with cardiogenic shock, and he was transferred to Coronary Unit on vasoactive drugs (Dobutamine and Norepinephrine). Later, the patient was stabilized and removed from orotracheal intubation and invasive mechanical ventilation and weaning from vasoactive drugs.

Laboratory tests revealed cardiac troponin I of 29.6 ng/mL (upper limit 0.1 ng/mL) and CK-MB peak value of 114 IU/L (reference value <25 IU/L). Transthoracic echocardiography showed moderate left ventricle systolic dysfunction caused by hypokinesia of both mid and basal of the anterior and septal segments, with mild dilatation of the ascending aorta.

 The patient denied having sexually transmitted disease or undergoing any specifically treatment. VDRL titers were 1/512, and FTA-ABS was reactive. Serologies for hepatitis B and C and HIV were not reagent. The analysis of the cerebrospinal fluid showed VDRL and indirect immunofluorescence not reagent. Treatment with benzathine penicillin in a dose of 1200000 IU/week by 21 days was started, and the patient was discharged asymptomatic on acetylsalicylic acid, clopidogrel, beta blockers, and angiotensin-converting enzyme inhibitor therapy.

## 3. Discussion

Syphilitic cardiovascular disease occurs more frequently than is recognized clinically [3]. Heggtveit [4] described that an accurate clinical diagnosis was established in only 17% of a large group of cases of syphilitic aortitis reviewed in a clinicopathological necropsy study. This manifestation should always be suspected especially in the paper of an ostial lesion on coronary angiography in a patient without major risk factors for atherosclerotic coronary artery disease [2]. In our case, the diagnosis of syphilitic aortitis with coronary ostium impairment was made due to high titers of VDRL, reflecting no treatment or reinfection, positive FTA-ABS, and angiographic findings including aortic regurgitation compatible with the disease.

In Brazil, the prevalence of syphilis remains relevant. Ferrari et al. [5] observed approximately 10.7% of congenital syphilis, considering newborn infants in necropsy study in Rio de Janeiro. Although patients frequently deny clinical antecedent of primary syphilis, Gomes [6] reported positive serology in 24.5% of individuals for this disease, considering prisoners population from the same city. Moreover, syphilis is an infectious disease occurring in sequential stages, remaining latent for several years [2] and in about 30% of the untreated patients, tertiary syphilis manifests between 10 to 30 years after the primary infection [7]. In this paper, screening for neurosyphilis and other sexually transmitted infections, including HIV, was too considered [8]. 

The cardiovascular manifestations of syphilis compromise asymptomatic aortitis, aortic regurgitation, coronary ostial stenosis, aortic aneurysm, and gummatous myocarditis [9]. Mostly, the syphilitic ostial stenosis is a disorder of the aorta rather than the coronary artery. In this paper, endarteritis obliterans of the vasa vasorum leads to medial necrosis with destruction of elastic tissue. Then, the syphilitic process extends to the ascending aorta, involving the orifices of the coronary arteries and producing ostial stenosis [10]. Extra ostial coronary artery syphilis occurs only rarely [4].

In our case, the manifestation of acute coronary syndrome with ST segment elevation required an emergency approach as the primary percutaneous coronary intervention. If the coronary artery bypass grafting was performed, the histopathological diagnosis could be obtained by aorta fragment showing inflammatory reaction with perivascular lymphocytic infiltrate compatible with syphilitic aortitis [2]. However, the absence of multiple risk factors for coronary artery disease in a younger individual, including hyperlipidemia, suggests that the atherosclerosis is unlikely in this patient [11]. 

Coronary ostial stenosis can be seen in approximately 26% of the patients with syphilitic aortitis [4], although it is uncommon for ostial lesion to lead to acute myocardial infarction [5, 12, 13]. The clinical presentation and the electrocardiographic findings in patients with syphilitic aortitis can be similar to individuals with atherosclerotic coronary artery disease [5]. However, while the treatment including revascularization is palliative in the second condition in patients with syphilitic coronary ostial stenosis it is healing [12]. In this paper, it is important to emphasize the etiology for inflammatory diseases including syphilis in the differential diagnoses for patients with coronary ostial lesion and normal distal bed, when the cure can be obtained through percutaneous coronary intervention and stenting [14].

Although no precedent for our patient, the radiation should be considered in the etiologic diagnosis for coronary artery obstructive disease involving ostial coronary segments and the left main [15, 16]. In this case, previous chemotherapy and radiation exposition exceeding 3000 Rad administrated on thorax should have been considered as important risk factors of radiation inducing heart disease [17, 18]. Moreover, isolated bilateral coronary ostial lesions because of aortoarteritis were mentioned by Lanjewar et al. [19], as well as lesions caused by Takayasu's aortitis and fibromuscular dysplasia have been described [9, 20, 21]. 

Recently, Machado et al. [2] described a case of 46-year-old patient with syphilitic aortitis admitted to the emergency department from our institution, with acute coronary syndrome submitted to fibrinolytic therapy and elective coronary artery bypass grafting. This paper showed a case of acute myocardial infarction caused by syphilitic coronary artery ostial stenosis treated with percutaneous coronary intervention and stenting, confirming that it is a possible and effective intervention in acute coronary syndrome emergency treatment. However, long-term followup of this patient is mandatory as a result of potential future aortic regurgitation and in-stent restenosis caused by continuous infection of the ascending aorta.
